# Engineering Lamellar Stainless Steel 410S Porous Supports via a Water-Based Tape Casting Method: A Scalable Path for MS-SOFCs

**DOI:** 10.1021/acsomega.5c05721

**Published:** 2025-10-29

**Authors:** João P. J. de Oliveira, Fabio C. Antunes, Victor C. Normandia, Thiago Dias, Reinaldo Cesar, Débora Vilela Franco, Leonardo Morais Da Silva, Gustavo Doubek, Hudson Zanin

**Affiliations:** 1 Advanced Energy Storage Division, Center for Innovation on New Energies, School of Electrical and Computer Engineering, University of Campinas, Av. Albert Einstein 400, Campinas, SP 13083-852, Brazil; 2 Centre for Energy and Oil Studies, University of Campinas, Av. Cora Coralina 350, Campinas, SP 13083-896, Brazil; 3 Advanced Energy Storage Division, Laboratory of Advanced Batteries (LAB), Center for Innovation on New Energies, School of Chemical Engineering, University of Campinas, Av. Albert Einstein 400, Campinas, SP 13083-852, Brazil; 4 Laboratory of Fundamental and Applied Electrochemistry, Department of Chemistry, Federal University of Jequitinhonha and Mucuri’s Valley, Rodovia MGT 367, km 583, 5000, Alto da Jacuba, 39100-000 Diamantina, MG, Brazil

## Abstract

Metal-supported solid oxide fuel cells (MS-SOFCs) have garnered increasing attention for advancing energy converter devices owing to their mechanical robustness, fast thermal cycling, and ability to operate with a wide range of fuels such as hydrocarbons and biofuels without contaminations. However, the manufacturing of porous metallic supports (PMSs), a critical component of these cells, still presents significant challenges, particularly regarding oxidation resistance, mismatch in thermal expansion coefficients (CTEs), gas diffusion issues, and synthesis reproducibility. In this work, we report a reproducible water-based tape casting methodology for PMS fabrication using commercially available 410S stainless steel powder. This alloy was selected due to their CTE compatibility with MS-SOFC ceramic layer components and due to its excellent chromium content (11.5–14.5%) to improve corrosion resistance. The optimized slurry formulation, containing tailored amounts of water-soluble binders and plasticizers, offers flexible and defect-free green tapes. Rheological characterization confirmed pseudoplastic and thixotropic behavior with high recovery (91.5%) after shear, ensuring stability during casting. Thermal gravimetric analysis (TGA) guided the debinding profile to prevent structural damage, and sintering was conducted under air and argon atmospheres. Argon sintering preserved the metallic structure and chromium content, while air sintering led to severe oxidation and phase destabilization. Precalcined ZrO_2_ nonstick coarse powder at 1600 °C for 2 h was used during sintering to prevent contamination from Al_2_O_3_. A well-developed lamellar microstructure and peculiar interconnected porosity were observed in the sintered PMS, both of which are fundamental for ensuring gas permeability in MS-SOFC applications. The permeability of the PMS was tested for H_2_, obtaining a result of 5.85 to 8.36 × 10^–12^ m^2^. Additionally, the PMSs showed an average resistivity of 2.45 ± 0.28 Ω·cm. This process addresses several obstacles in PMS fabrication pathway for integrating PMSs into next-generation SOFC architectures.

## Graphical abstract

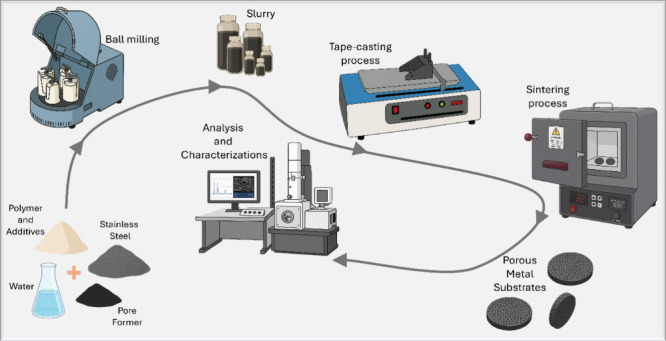

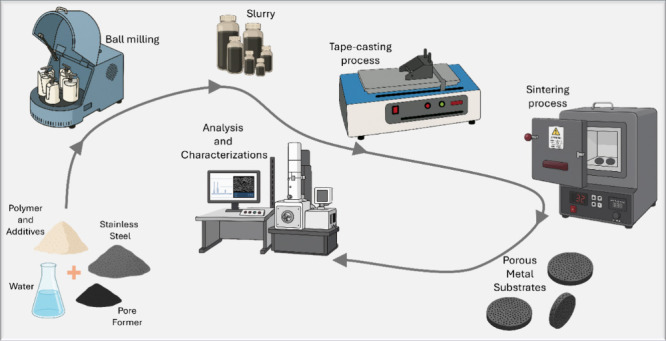

## 1 Introduction

Solid oxide fuel cells (SOFCs) are high-temperature electrochemical devices that convert chemical energy into electricity with exceptional efficiency and minimal environmental impacts.^1^ Their fuel flexibility, ranging from hydrogen (H_2_) to various hydrocarbon compounds, makes them attractive for both stationary and mobile energy applications.^2–4^ Despite their efficiency, conventional ceramic-supported SOFCs remain limited by intrinsic brittleness, long startup times, and high fabrication costs, all of which constrain their scalability and broader adoption.^5^ MS-SOFCs have been proposed as a robust replacement to overcome the mechanical and economic limitations of conventional SOFCs.^2^ By replacing brittle ceramic scaffolds with metallic supports, typically ferritic stainless steels or Ni-based alloys, MS-SOFCs combine enhanced mechanical strength, faster thermal response, and lower production costs. These attributes are particularly advantageous for dynamic applications, such as transportation, where thermal cycling and startup time are critical. As noted by Tucker^6^ and others,^7,8^ the metallic scaffolds improve manufacturability and enable more flexible system integration. Moreover, innovations such as porous Ni–Fe alloy supports have improved redox stability and CTE across cell layers.^9^ Nevertheless, this technology still faces unresolved barriers: high-temperature oxidation of metallic supports, interfacial delamination due to CTE mismatches, and the need for scalable, defect-free fabrication routes for PMSs.^2,10^

Tape casting stands out among the available manufacturing methods for PMSs in MS-SOFCs due to its simplicity, versatility, and cost-effectiveness.^6,8^ Unlike techniques such as uniaxially and isostatic pressing or laser drilling metallurgy, tape casting is a kind of powder metallurgy conformation that enables fine control over the support thickness, porosity, and composition, making it especially suitable for producing uniform and thin PMSs at a scale manufacturing level.^11^ The process involves spreading a tailored slurry onto a polymeric substrate using a doctor blade, followed by controlled drying and sintering. Advances in slurry formulation, particularly in rheology, dispersion stability, organic additive tuning, and optimized thermal treatment profiles, have significantly improved the structural quality and functional performance of PMS components. As a result, tape casting has become a leading candidate for the scalable manufacturing of PMSs for MS-SOFCs.^12^

Recently, de Oliveira et al.^8^ conducted an extensive study highlighting the importance of each material used in PMS suspensions for tape casting. In that work, the authors demonstrated that PMS suspensions face a significant challenge in particle stabilization due to the high density of metallic powders, thus selecting the appropriate dispersant is crucial to achieve effective particle stabilization.^13,14^ Beyond dispersants, the formulation of suspensions for tape casting generally includes binders and plasticizers, which also contribute to the colloidal stability of the system.^15^ However, the role of these additives extends beyond mere stabilization. In particular, the binder plays a fundamental role in forming bridges between particles, maintaining the structural integrity of the tape during solvent evaporation and ensuring proper cohesion of the green body.^16,17^ Plasticizers, in turn, are added to reduce the glass transition temperature of the main binder, promoting the transition from a vitreous to a more ductile state, thereby improving the flexibility of the green tape without compromising its structural integrity during handling and sintering.^18^ Moreover, since PMS produced by tape casting must be porous to allow gas transport during MS-SOFC operation, many formulations incorporate pore formers to create interconnected channels that enable fuel to reach the anode for electrochemical reactions to occur. In general, several strategies have been employed to obtain PMS structures with properties suitable for MS-SOFC applications, which often involve the use of different dispersants, binders, plasticizers, and pore formers.^8,16,19–24^

Another important point to highlight is that aqueous tape casting is preferable to the nonaqueous route due to its significant environmental, scalability, and safety advantages, as it relies on nontoxic systems, making it a sustainable alternative to organic-based formulations.^8,25^ These aqueous systems, which employ low-cost water as the solvent, eliminate the need for special precautions against toxicity or flammability and avoid the implementation of solvent recovery systems.^8,25,26^ However, the aqueous process is considerably more challenging because the formulations are complex multiphase systems that are extremely sensitive to variations in processing conditions, particularly in terms of drying parameters and slurry composition.^26^ Aqueous suspensions generally exhibit inferior drying characteristics, such as a high latent heat of evaporation, and are prone to defects including trapped air bubbles and agglomerates, which can lead to reduced green and final densities.^26^ The difficulty is further compounded by the fact that many aspects of aqueous tape casting remain insufficiently understood, and the selection of additives is still largely based on empirical observations rather than a comprehensive understanding of the underlying physicochemical mechanisms.^27^

Despite its advantages, tape casting still poses critical challenges when applied to the fabrication of PMSs for MS-SOFCs. Achieving uniform tape thickness and controlling the porosity distribution after sintering are essential yet difficult tasks. Common defects, such as cracking, delamination, and warping, can arise during drying, debinding, or thermal treatment, compromising the mechanical integrity and reproducibility of the PMSs. Furthermore, strong adhesion between the PMS and subsequent cell layers is vital for long-term stability but is often hindered by residual stresses or interfacial mismatches.^8^ Equally complex is the optimization of slurry composition: balancing the metallic content, binders, and pore structures to ensure both electrochemical performance and mechanical robustness requires meticulous formulation and detailed process control.^2,5,8,10^

In this study, we present a reproducible and scalable procedure for fabricating PMSs via aqueous tape casting, employing 410S stainless-steel powder. This alloy was selected for its CTE range between 9.9 and 11.7 × 10^–6^ °C^–1^ in the temperature range of 100–538 °C,^28^ which closely matches that of typical MS-SOFC ceramic components, thus minimizing interfacial stress during thermal cycling.^6^ The formulation of the slurry was carefully optimized, particularly the concentrations of binders and plasticizers, to yield flexible green tapes free of visible defects. Rheological characterization was performed to ensure high suspension stability and casting reliability. The sintering process was systematically investigated under both oxidizing and inert atmospheres, with process parameters tailored to improve reproducibility and minimize contamination. Notably, substrates sintered in argon exhibited minimal oxidation, positioning this approach as a viable alternative compared to more complex reducing environments commonly reported in the literature.^20,21,29–33^ The final PMSs displayed a lamellar microstructure with high porosity, likely resulting from the synergistic effect of pore architectures and the presintering lamination step, both essential features for gas diffusion and mechanical functionality in MS-SOFCs.

## 2 Experimental Procedure

### 2.1 Sample Preparation

PMSs were fabricated via tape casting using commercial SS 410S powder (GoodFellow). The slurry was formulated in deionized water and included the following additives: ammonium polyacrylate (Miracema-Nuodex) as a dispersant, poly­(vinyl alcohol) (Dinâmica) as the primary binder, poly­(acrylic acid) (Sigma-Aldrich) as a cobinder, triethylene glycol (Sigma-Aldrich) as a plasticizer, Triton X-100 (Sigma-Aldrich) as a surfactant, and CB2000BP carbon black (Cabot) as the pore-forming agent. To prevent bubble formation, *n*-octyl alcohol (Sigma-Aldrich) was added as an antifoam agent. Slurry homogenization was carried out via the ball milling process for 72 h using 3Y-TZP grinding balls in a Nalgene bottle. Following milling, the suspension underwent an aging step under mechanical stirring at 300 rpm and 50 °C for 24 h to stabilize viscosity.

Rheological characterization was performed using an MCR 92 rheometer (Anton Paar) with a 25 mm parallel plate-cone geometry and a 1 mm gap. The suspension was then cast onto a Mylar substrate using a doctor blade apparatus set to 1 mm height. After room-temperature drying, the green tape was laminated using a hot roller press (TOB Machine) under the following conditions: 600 μm gap, 1 mm s^–1^ speed, and 60 °C. Discs with 32 mm diameter were subsequently punched from the laminated tape and sintered under controlled inert (argon) and oxidizing (air) atmospheres.

To evaluate the effect of organic additive content on tape quality, three different slurry formulations were prepared, varying the relative amounts of binder, cobinder, and plasticizer. The volumetric percentages of the binder and plasticizer used in these formulations are summarized in Table 1. The ratio between binder, cobinder, and plasticizer was adjusted progressively to assess the impact on green tape formation, flexibility, and defect occurrence. This formulation recipe was used as the basis for selecting the optimal composition for subsequent rheological and sintering analyses. An overview of the volumetric quantities of each component in suspensions is shown in Table S1.

**Table 1:** Values of the Amount of Binder and Plasticizer Referring to Figure 2a–d

**component**(% vol)	**tape A**	**tape B**	**tape C**	**tape D**
binder	11.9	15.6	18.3	22.5
cobinder	4.9	5.2	7.6	11.2
plasticizer	10.7	13.1	16.5	20.7
binder:cobinder:plasticizer ratio	2.4:1:2.2	3:1:2.5	2.4:1:2.2	2.0:1:1.8

### 2.2 Characterization Studies

The microstructural and physicochemical properties of the sintered PMSs and raw powders were characterized using different techniques. Morphological surface analysis was performed by scanning electron microscopy (SEM) using a Thermo Quattro ESEM (Thermo Fisher, USA), equipped with secondary electron (SE), backscattered electron (BSE), and energy-dispersive X-ray spectroscopy (EDS, Ultradry model) detectors. These detectors were employed to evaluate the particle morphology of the SS 410S powder, as well as the surface and fracture cross sections of PMS samples sintered under inert and oxidizing conditions. Phase composition and crystallographic structure were examined via X-ray diffraction (XRD) using a D2 Phaser diffractometer (Bruker, Germany) operated in Bragg–Brentano geometry over a 2θ range of 20° to 90°, with a step size of 0.05°, an acquisition time of 0.5 s per step, and a sample rotation speed of 5 rpm. The instrument was equipped with a LYNXEYE TM linear detector and a Cu–K_α1_ radiation source (λ = 1.5406 Å and 8.047 keV). Particle size distribution of the SS 410S powder was assessed by laser diffraction using a Mastersizer 3000 (Malvern Instruments, UK), and relevant statistical parameters (D10, D50, D90, and span) were evaluated. The specific surface area of the powder was determined by nitrogen adsorption–desorption isotherms at 77 K using the BET method on a Micro 100 surface area analyzer (JWGB Instruments, China). TGA was performed using a STA 449 F5 Jupiter (Netzsch, Germany) to determine the organics' thermal decomposition to define the optimal debinding profile. Measurements were carried out in synthetic air from 25 to 800 °C using an Al_2_O_3_ crucible and a heating rate of 5 °C min^–1^. Porosity and pore size distribution of sintered samples were quantified via mercury intrusion porosimetry (AutoPore IV, Micrometrics, USA), with applied pressures ranging from 0.5 to 60,000 psi.

Electrical characterization was performed using a parameter analyzer model Keithley 4200-SCS (Keithley Instruments, USA) with direct current (DC) over a potential range of −1.0 to 1.0 V. The resistance of the samples was calculated from the conductance obtained from the slope of the current–voltage (*I*–*V*) curves. Mechanical tests were conducted using a Texture Analyzer universal testing machine (Model TA500, Lloyd Instruments, England) equipped with a maximum load cell capacity of 500 N. The specimen was supported on a square, hollow base with a central aperture of 12.5 mm, and the samples were precisely centered over this hole. Furthermore, a conical indenter with a 30° tip angle was utilized to apply the load. In addition, the tests were performed at a crosshead speed of 1 mm min^–1^, and a preload of 0.1 N was applied to the specimen at the beginning of the analysis.

H_2_ permeability analysis was carried out using the setup shown in Figure S1. The setup inspired in Furumoto et al.^34^ consists of two Inconel flanges with gas inlet and outlet, where the sample was placed between the flanges and sealed with plastic silicone. The H_2_ gas flow was controlled by a Horiba Mass Flow Controller connected to a HUB Controller. The pressure difference between the inlet and outlet of the system was measured using a pressure transducer. Permeability (*k*) was calculated according to Darcy’s law (eq 1):

$$k=\frac{Q\mu L}{A\Delta P}$$ 1

where *Q* is the applied gas flow rate, μ is the viscosity of H_2_, *L* is the sample thickness, *A* is the cross-sectional area of the SS 410S PMS, and Δ*P* is the pressure difference between the gas inlet and outlet of the system.

## 3 Results and Discussion

### 3.1 Powder Characterization

Although the stainless-steel powder used for PMS fabrication is commercially available, thorough characterization is essential to ensure its suitability for tape casting applications. For this purpose, the SS 410S powder was analyzed by SEM, XRD, laser diffraction, and BET surface area. SEM imaging (Figure 1a) revealed a heterogeneous morphology composed of spherical and elongated particles with varying sizes. The XRD pattern (Figure 1b) confirmed the presence of a body-centered cubic (BCC) α-FeCr solid solution phase, with diffraction peaks at 2θ values of 44.6° (0 1 0), 64.9° (0 2 0), and 82.2 (1 2 1), in agreement with reference data (COD, PDF #96–152–3983)^35^ and prior reports.^36,37^ Particle size distribution, measured via laser diffraction in dry mode using compressed air at 2 bar and a feed rate of 50%, is shown in Figure 1c. The results, averaged over five measurements, indicate a size range between 5 and 100 μm, with the majority of the volume fraction distributed between 15 and 75 μm. While SEM images suggest some irregularities in particle shape and size, the overall distribution aligns well with the literature for tape casting applications.^38^ Surface area analysis via nitrogen adsorption at 77 K yielded a specific surface area of approximately 1.2 m^2^ g^–1^ (Figure 1d), which is consistent with previously reported values for ferritic stainless-steel powders.^37,39,40^ The corresponding adsorption–desorption isotherm exhibits a Type II profile,^41^ characteristic of nonporous or macroporous materials, further confirming the low surface area typically associated with dense metallic particles.

**Figure 1:**
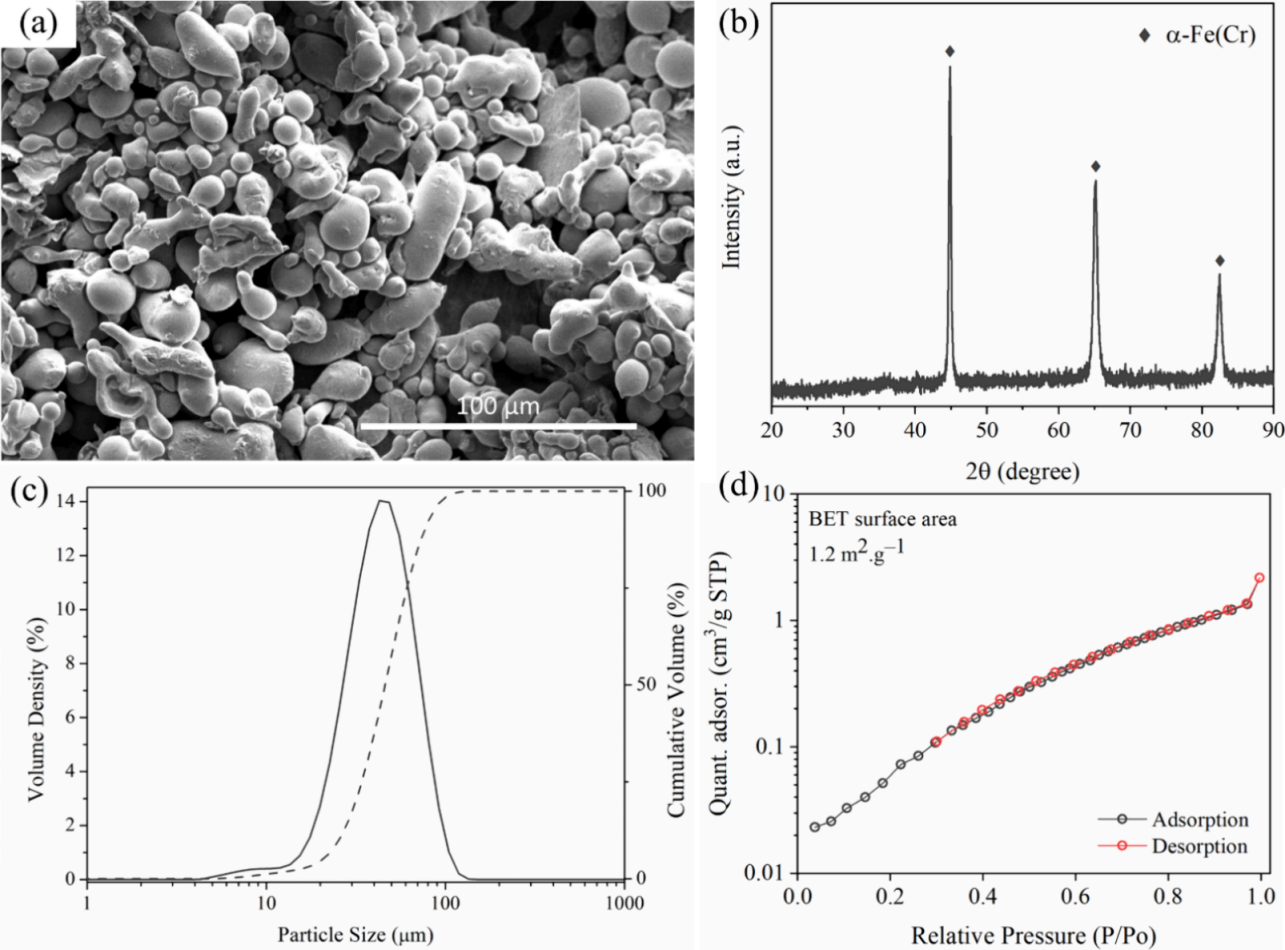
Characterization of the SS 410S powder used for PMS fabrication: (a) SEM image showing heterogeneous morphology with spherical and elongated particles; (b) XRD pattern confirming the α-Fe­(Cr) phase with a BCC structure; (c) particle size distribution obtained by laser diffraction, indicating a dominant range between 15 and 75 μm; (d) N_2_ adsorption–desorption isotherm at 77 K, displaying a Type II profile and a BET specific surface area of 1.2 m^2^ g^–1^.

Table 2 summarizes the parameter values obtained from laser diffraction analysis of the SS 410S stainless steel powder. The particle size distribution is relatively narrow and symmetric, as indicated by the D10, D50, and D90 values. The D10 value of 23.8 μm implies that 10% of the particles are smaller than this size, while the D50 (median diameter) of 43.3 μm represents the midpoint of the distribution. The D90 value of 72.1 μm shows that 90% of the particles are below this size, indicating that most of the powder lies within the 23–72 μm range. The calculated span (i.e., grade of uniformity) of 1.12 suggests good distribution uniformity, which is essential for stable tape casting behavior and homogeneous microstructure formation during sintering.^42^

**Table 2:** Statistical Parameters of the Particle Size Distribution of SS 410S Powder Obtained by Laser Diffraction Analysis

**parameters**	**SS 410S**
D10 (μm)	23.82 ± 0.18
D50 (μm)	43.29 ± 0.15
D90 (μm)	72.08 ± 0.16
span	1.116 ± 0.005

### 3.2 Tape Casting and Slurry Optimization

The quality of green tapes produced via tape casting is highly sensitive to the proportions of binder and plasticizer used in the slurry formulation. Figure 2 illustrates the tapes cast from aqueous slurries with varying binder and plasticizer contents (see Table 1). Tapes A and B (Figure 2a and b) exhibit evident surface defects, including cracks and irregularities, which may arise from multiple factors such as inadequate binder content, insufficient plasticization, suboptimal milling time, or poor particle dispersion. In this experiment, however, the only parameter intentionally varied were binder and plasticizer concentrations. As expected, low additive content hindered proper particle rearrangement and green compaction within the tape matrix, promoting crack formation during drying.^43^ With progressive increases in binder and plasticizer concentrations, the visual quality of the tapes improved significantly. Tapes C and D (Figure 2c and d), cast with the highest amount of additives, displayed a uniform and defect-free surface with good flexibility and mechanical integrity. However, Tape D, after drying on the Mylar substrate, showed signs of warping. This phenomenon may occur due to various factors, one of which could be the excessive amount of polymeric binder added.^30^ Such warping can lead to heterogeneous particle packing in certain regions of the tape, which may subsequently impair the sintering process, resulting in cracks and deformation of the parts. Due to its superior appearance and handling characteristics, the formulation used for tape C was selected for further rheological and sintering studies.

**Figure 2:**
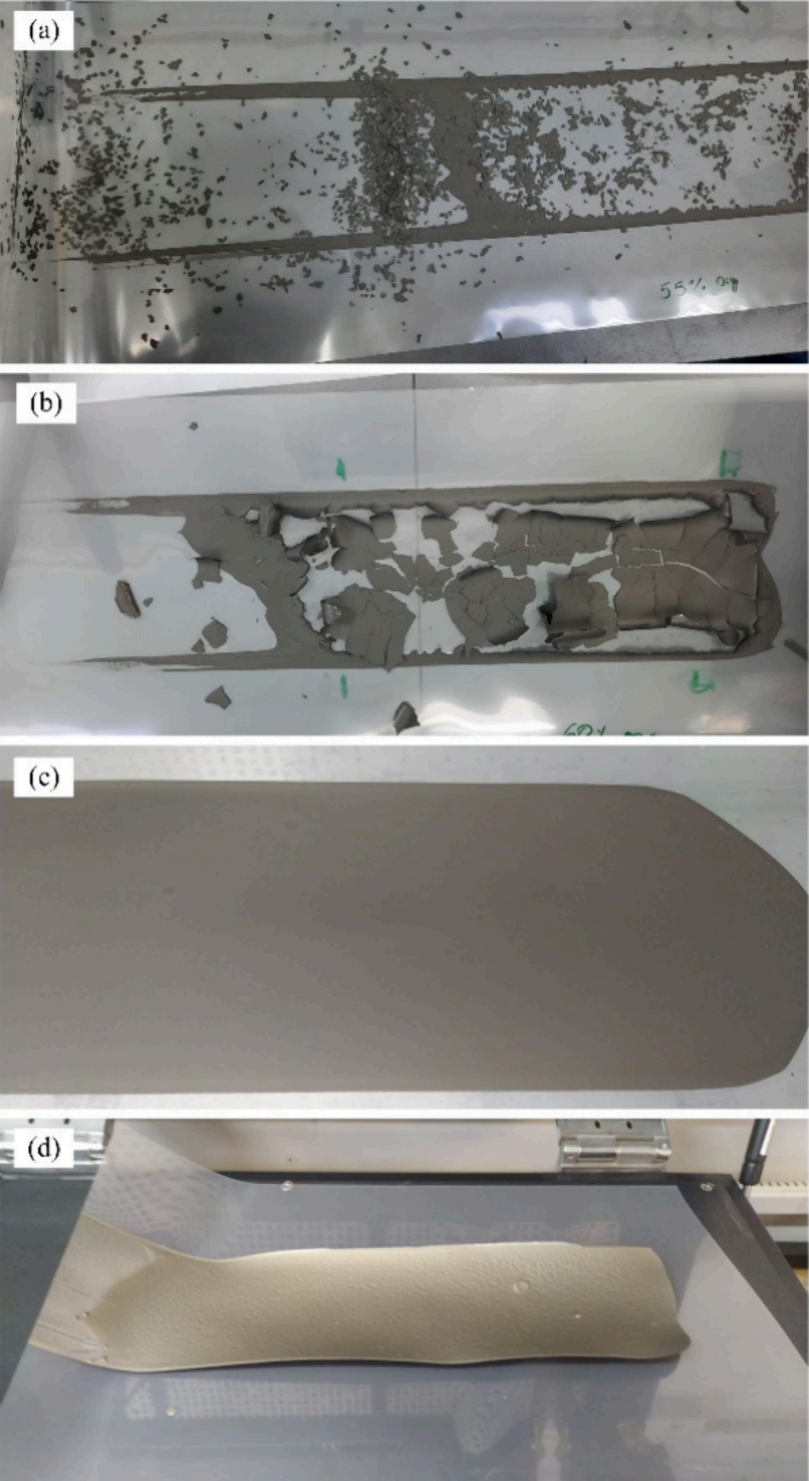
Tapes with different amounts of binder and plasticizer: (a) Tape A, (b) Tape B, (c) Tape C, and (d) Tape D.

### 3.3 Rheological Study for the Suspension

Rheological analyses were conducted for the suspension used to produce Tape C and Tape D (Supporting Information), since the latter was the formulations that yielded visually uniform and defect-free tapes. The other slurries were excluded from rheological testing due to their insufficient fluidity, likely caused by a suboptimal concentration of organic additives that could have damaged the cone–plate rheometer setup. The measurements were performed at 25 °C using a 25 mm cone–plate geometry with a fixed gap of 1.0 mm. Figure 3a shows the viscosity profile of the Tape C suspension as a function of shear rate. The suspension exhibited non-Newtonian, pseudoplastic behavior, characterized by a progressive decrease in viscosity with an increasing shear rate.^44^ At a shear rate of 10 s^–1^, considered ideal for casting, a distinct inflection point is observed, with viscosity ranging between 1800 and 2000 mPa s, indicating appropriate flowability during casting. The Tape D suspension has the same profile (Figure S2a); however, the viscosity value is much higher. Figure 3b presents the stability test under constant shear (50 s^–1^) for 120 s, a condition that exceeds the typical tape deposition time. The suspension maintained a stable viscosity throughout the test, confirming its suitability for tape casting operations that demand consistent flow properties. The suspension of Tape D showed lower stability (Figure S2b), as its viscosity continued to decrease after 120 s. To further investigate the thixotropic behavior, a three-interval thixotropy test (3iTT) was performed (Figure 3c). In the first interval, the suspension was subjected to low shear (1 μN m) for 120 s, stabilizing at ∼3780 mPa s. In the second interval, a high shear torque (1500 μN m) was applied, causing the viscosity to drop sharply to ∼228 mPa s, indicating structural breakdown. In the final interval, the system was allowed to rest at 1 μN m, and viscosity recovered to ∼91.5% of its initial value, reaching ∼3484 mPa s over 540 s. This recovery confirms the thixotropic nature of the suspension and further supports its classification as a pseudoplastic fluid. The suspension of Tape D did not show any recovery; in fact, it exhibited further deterioration (Figure S2c). This behavior appears to be related to its high content of organics and powder, which results in a lower fraction of solvent. When subjected to very high torque, the viscosity rises to extreme levels, making it impossible to ascertain the accuracy of the measured value.

**Figure 3:**
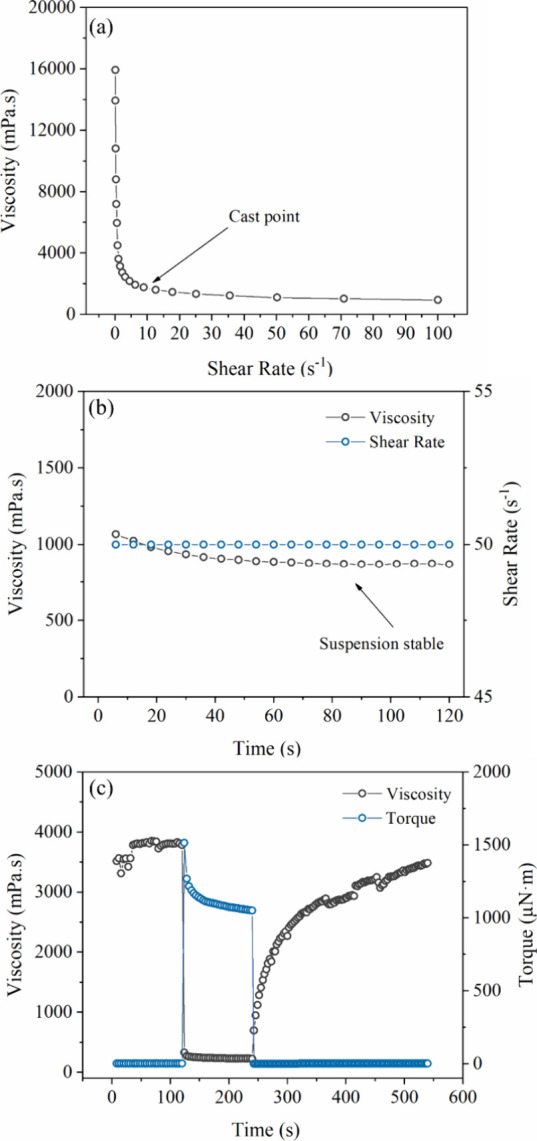
Rheological behavior of the optimized PMS suspension used for Tape C fabrication: (a) viscosity profile as a function of shear rate at 25 °C, showing pseudoplastic behavior and identifying the casting shear rate (∼10 s^–1^); (b) viscosity stability under a constant shear rate of 50 s^–1^ for 120 s, simulating the tape casting deposition time; (c) three-interval thixotropy test (3iTT), showing viscosity recovery (∼91.5%) after structural breakdown caused by high shear torque (1500 μN m), confirming thixotropic behavior.

### 3.4 Thermogravimetric Analysis and Sintering Parameters

Monitoring the debinding process is essential to ensure the integrity and quality of the final ceramic part. To this end, TGA provides crucial insights into the removal behavior of organic binders. Figure 4 shows the TGA and differential thermogravimetric (DTG) curves for the green ceramic tapes prepared with composition C, conducted in a synthetic air flow rate. An overall weight loss of approximately 35% was observed, corresponding to the combustion of organic compounds up to around 600 °C. The TGA/DTG profile reveals four distinct regions of mass loss. The first region (50–175 °C) corresponds to the evaporation of adsorbed water and water weakly bound to the polymer matrix formed by binders and plasticizers.^45^ The second region (220–360 °C) shows significant mass loss due to the decomposition and carbonization of the PVA binder and TEG plasticizer.^45^ The third region (410–490 °C) is attributed to the degradation of the dispersant and PAA cobinder, involving the breakdown of C–O, C=O, and C–C bonds in the polymer backbone.^46,47^ The fourth region (550–610 °C) is associated with the burnout of the pore-forming agent (carbon black) and potentially with the CO_2_-related degradation phenomenon.^48^ The controlled removal of these organics, commonly referred to as the debinding step, is particularly critical in tape-cast green tapes due to their high polymer content. Improper burnout can lead to residual carbon, microstructural defects, delamination, and even catastrophic cracking or part disintegration. While faster heating rates accelerate the combustion of organics, they also increase the risk of defects in the green body.^49–51^ Thus, a slow and controlled heating ramp is essential. Literature recommends heating rates of 0.5–1.0 °C min^–1^ for the organic burnout stage and 2.0–3.0 °C min^–1^ for reaching the sintering temperature.^52,53^ In this study, a heating rate of 0.6 °C min^–1^ was employed up to 600 °C, followed by a 1 h residence time to ensure complete organic removal, as supported by the final inflection in the TGA/DTG curves. For the sintering step, a rate of 3.0 °C min^–1^ was applied up to 1300 °C, with a 2 h residence time, avoiding temperatures above 700 °C in an oxidizing atmosphere to prevent the PM severe oxidation.

**Figure 4:**
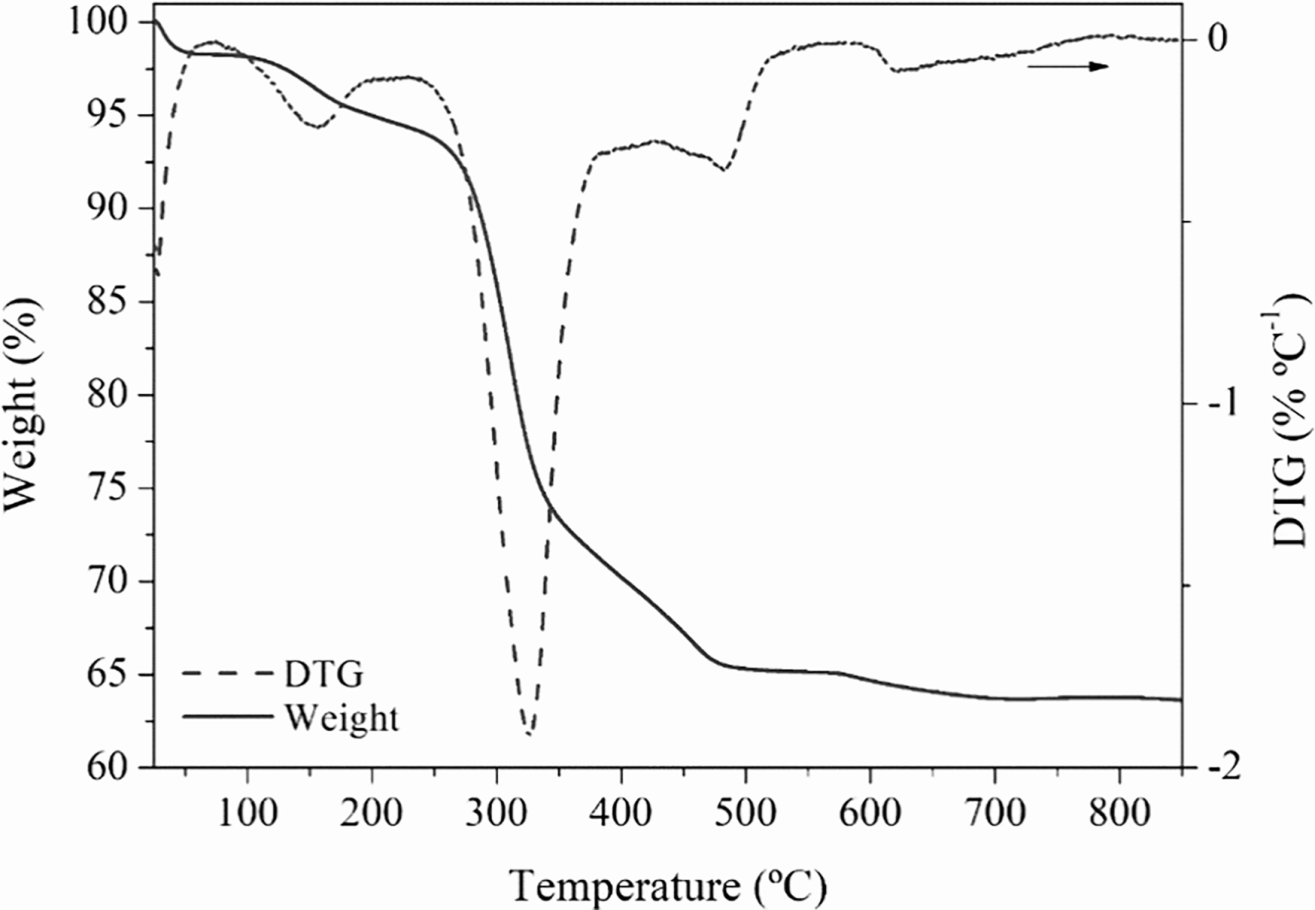
TGA and DTG of green Tape C in synthetic air. The TGA curve (solid line) indicates a total weight loss of approximately 35%, associated with the thermal decomposition of organic components during the debinding process. The DTG curve (dashed line) reveals at least three main decomposition events: (i) moisture evaporation and removal of weakly bound water below ∼175 °C, (ii) major degradation of polymeric binders and plasticizers at 220–360 °C, and (iii) gradual degradation of residual organics and carbonaceous matter from 410 °C up to ∼600 °C. Thermal stability is observed to be above 650 °C.

As discussed earlier, the debinding and sintering stages are critical for achieving ceramic components with adequate mechanical integrity and microstructural homogeneity. One of the main challenges during sintering is preventing sample warping, which can arise from gradients in organic content, layer thickness, or temperature. To mitigate such defects, several studies have recommended the use of refractory materials as sintering loads.^22,54,55^ In this study, we evaluated the effectiveness of porous Al_2_O_3_ refractories in minimizing warping during the sintering of PMS samples. Additionally, a precalcined ZrO_2_ powder was employed to avoid direct contact between the green samples and the refractory surface. Figure 5 illustrates the impact of different sintering setups on sample integrity. In Figure 5a, a PMS sample sintered without any load shows pronounced warping due to unconstrained shrinkage. Figure 5b reveals severe degradation and fragmentation of the sample, with blackened residues and fractured fragments adhered to the alumina surface, suggesting a chemical reaction or thermal incompatibility between the sample and refractory. Figure 5c shows a PMS sample sintered between two alumina supports, where the sample remains physically intact but displays surface contamination due to direct interaction. To resolve this, a thin layer of precalcined ZrO_2_ powder was applied between the sample and refractory. As seen in Figure 5d, this barrier successfully prevented chemical interaction and preserved the integrity of the PMS surface, with no visible contamination. The PMS samples of Tape D exhibited the same behavior during sintering, as shown in the optical images in Figure S3a–c.

**Figure 5:**
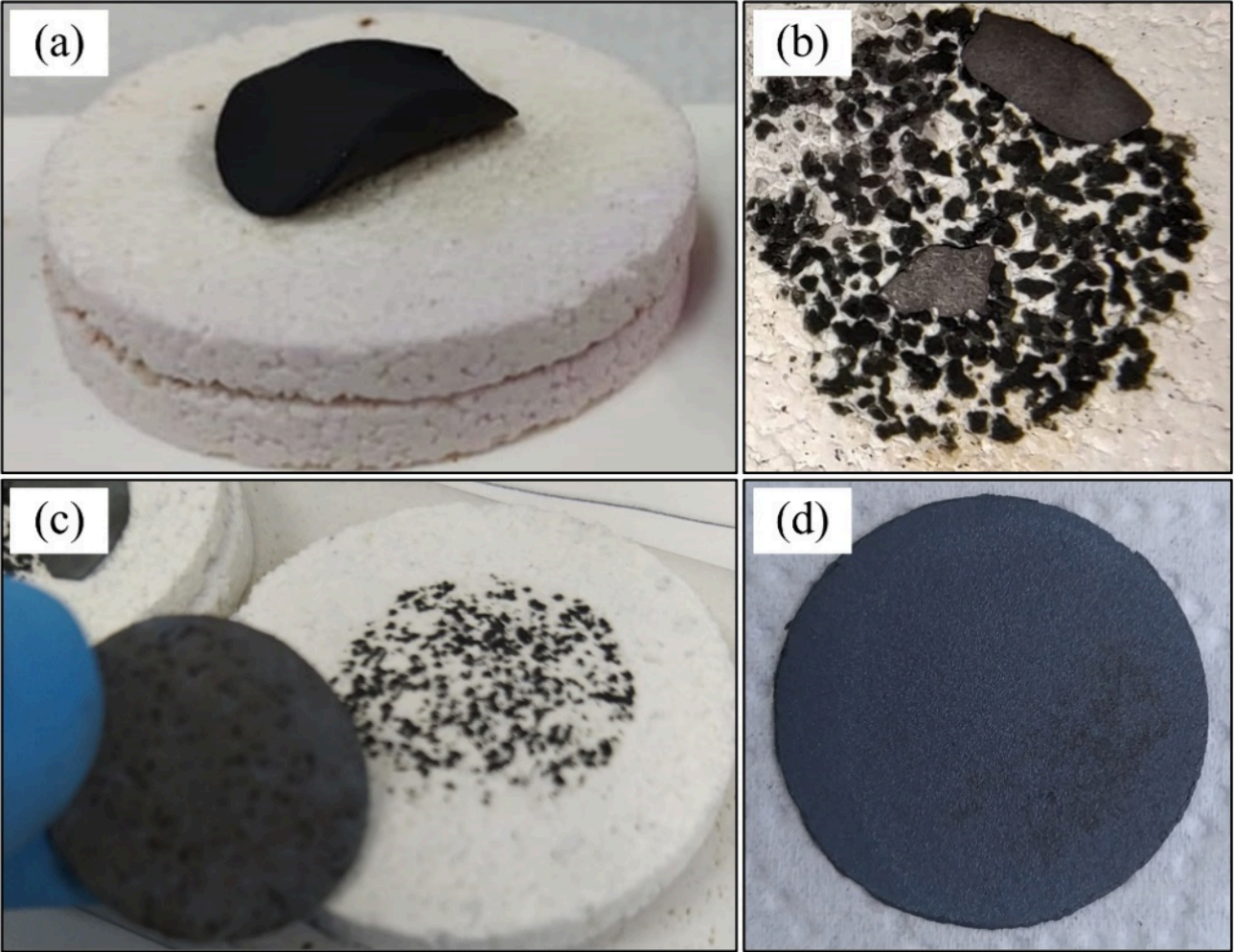
Photographs illustrating the effect of sintering conditions on PMS samples. (a) Presintered PMS sample without sintering load, showing severe warping due to unconstrained shrinkage. (b) PMS fragments adhered to the alumina refractory surface, indicating chemical interaction and sample degradation. (c) Intact PMS sample sintered between alumina refractories, exhibiting surface contamination (black spots) caused by interaction with the refractory. (d) PMS sample sintered between refractories with a precalcined ZrO_2_ powder, showing no visible warping or contamination, confirming the effectiveness of the protective layer.

In summary, when sintering loads are not used, the sample remains unconstrained during debinding and sintering, which can lead to warping due to gradients in powder or binder content, thickness irregularities, or temperature variations during heating. These factors are known to cause defects such as warping, as well-documented in the literature.^54,55^ Therefore, the use of sintering loads is recommended to obtain flat, defect-free components. However, since direct contact between sintering loads and PMS materials may result in chemical reactions or restrict shrinkage, it is strongly advised to use a protective ZrO_2_ powder layer as an intermediate barrier.

The linear shrinkage of PMS samples sintered at 1300 °C was evaluated under two conditions: (i) without the use of a sintering load and (ii) with the application of a sintering load combined with a precalcined ZrO_2_ powder to prevent direct contact between the sample and the refractory surfaces. Although warped samples are unsuitable for practical use as PMS components, their analysis provides valuable insight into the maximum unconstrained shrinkage during sintering. Table 3 presents a comparison of the average shrinkage values for PMS samples sintered with and without a 14 g sintering load and precalcined ZrO_2_ powder layer in an inert atmosphere with a 4000 sccm argon flow rate. The results show that the use of a sintering load effectively suppresses warping by constraining lateral shrinkage and promoting densification along the thickness direction. These findings are consistent with the observations in Figure 5a–d and the associated discussion. The sintering results of Tape D samples with sintering load and ZrO_2_ powder layer are presented in Table S2. It can be observed that the sintered Tape D samples exhibited greater shrinkage in diameter and lower shrinkage in thickness compared with those of Tape C. This indicates that changing the amount of organics in the suspensions can affect the way the polymer organizes the metallic particles. Despite the significant shrinkage in thickness, the samples sintered with the load exhibited good mechanical strength, which is particularly important for subsequent handling and deposition of functional layers in MS-SOFC fabrication.

**Table 3:** Linear Shrinkage Parameters of PMS Samples of Tape C Sintered at 1300 °C in an Argon Atmosphere under Different Conditions: (i) without Sintering Load and (ii) with Sintering Load plus Precalcined ZrO_2_ Powder Layer^a^

**parameters** (*n* = 3 **samples)**	**average size without sintering load**	**shrinkage without sintering load (%)**	**average size with sintering load and precalcined ZrO** **_2_** **powder layer**	**shrinkage with sintering load and precalcined ZrO** **_2_** **powder layer (%)**
diameter	27.9 ± 0.16 mm	13.4 ± 0.19	29.6 ± 0.22 mm	8.6 ± 0.73
thickness	656 ± 33.8 μm	5.7 ± 5.0	460.0 ± 32.9 μm	31.1 ± 0.69

a Values represent the average shrinkage percentage in diameter and thickness directions of three different SS 410S PMS samples (*n* = 3 samples).

### 3.5 Morphological Characterization Studies

The surface and cross-sectional morphology of the PMS was analyzed by SEM to better understand the microstructural evolution after sintering. Microstructural features such as pore distribution, grain boundaries, and potential alloying elements and impurity segregation are critical in assessing gas transport efficiency and ohmic losses in fuel cells. At the same time, the formation of secondary phases due to segregation may obstruct electronic conduction pathways, negatively impacting PMS performance. Figure 6 presents the SEM micrographs at different magnifications of the fracture cross-section and surface of a PMS sample sintered at 1300 °C for 2 h under a 4000 sccm argon flow rate. In Figure 6a, the low-magnification cross-section reveals a lamellar microstructure with an overall thickness of approximately 600 μm, characteristic of the tape casting process. Figure 6b, at higher magnification, shows the internal porosity more clearly, with pores ranging from 10 to 100 μm dispersed between the lamellae structure. Figure 6c displays the sintered surface, where ferritic grains of SS 410S exhibit well-defined grain boundaries with intergranular pores and interconnected pore channels. Finally, Figure 6d provides a higher magnification view of the surface, from which an average grain size of 2.6 μm was estimated. These morphological characteristics indicate a porous, interconnected structure suitable for applications requiring gas permeability, such as in MS-SOFC supports.

**Figure 6:**
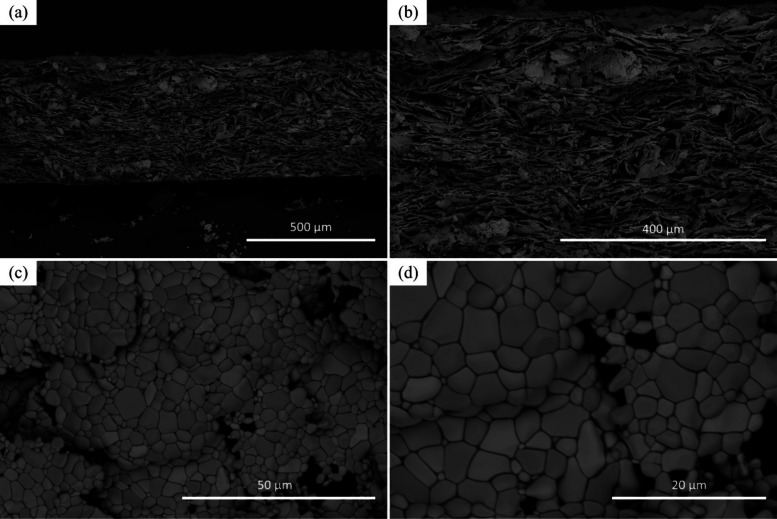
SEM micrographs of PMS SS 410S sintered at 1300 °C for 2 h under an argon atmosphere (4000 sccm). (a, b) Fracture cross sections at different magnifications, highlighting the lamellar structure and porosity; horizontal field widths (HFW): 1500 μm (a) and 750 μm (b). (c, d) Surface micrographs showing ferritic grain structure with well-defined grain boundaries and intergranular porosity. HFW: 100 μm (c) and 50 μm (d).

A detailed pore size analysis of sintered SS 410S PMS was performed using mercury intrusion porosimetry. As shown in Figure 7a, the incremental intrusion curve reveals a small peak between 1 and 2 μm, followed by a pronounced peak in the 1–6 μm interval and a broader plateau extending from 10 to 100 μm.^56^ This profile indicates a multimodal pore size distribution, with dominant interstitial pores ranging from 1 to 8 μm and larger interconnected macropores in the 20–80 μm interval.^57^ The high cumulative porosity, despite sintering at elevated temperature, is attributed to the intentional addition of pore formers during processing. The interstitial porosity was measured at 47.6%, while the total porosity reached 66.5%. Additionally, the apparent permeability measured with liquid mercury was 3.26 × 10^–12^ m^2^. The apparent and skeletal densities were determined to be 1.88 g cm^–3^ and 5.64 g cm^–3^, respectively. Figure 7b presents the mercury intrusion–extrusion curves, highlighting a total intrusion volume of 0.353 cm^3^ g^–1^ and an extrusion volume of 0.338 cm^3^ g^–1^. The minimal hysteresis observed indicates limited pore closure and good accessibility of interconnected pores, with a small fraction of mercury retained within the structure after depressurization.^58^

**Figure 7:**
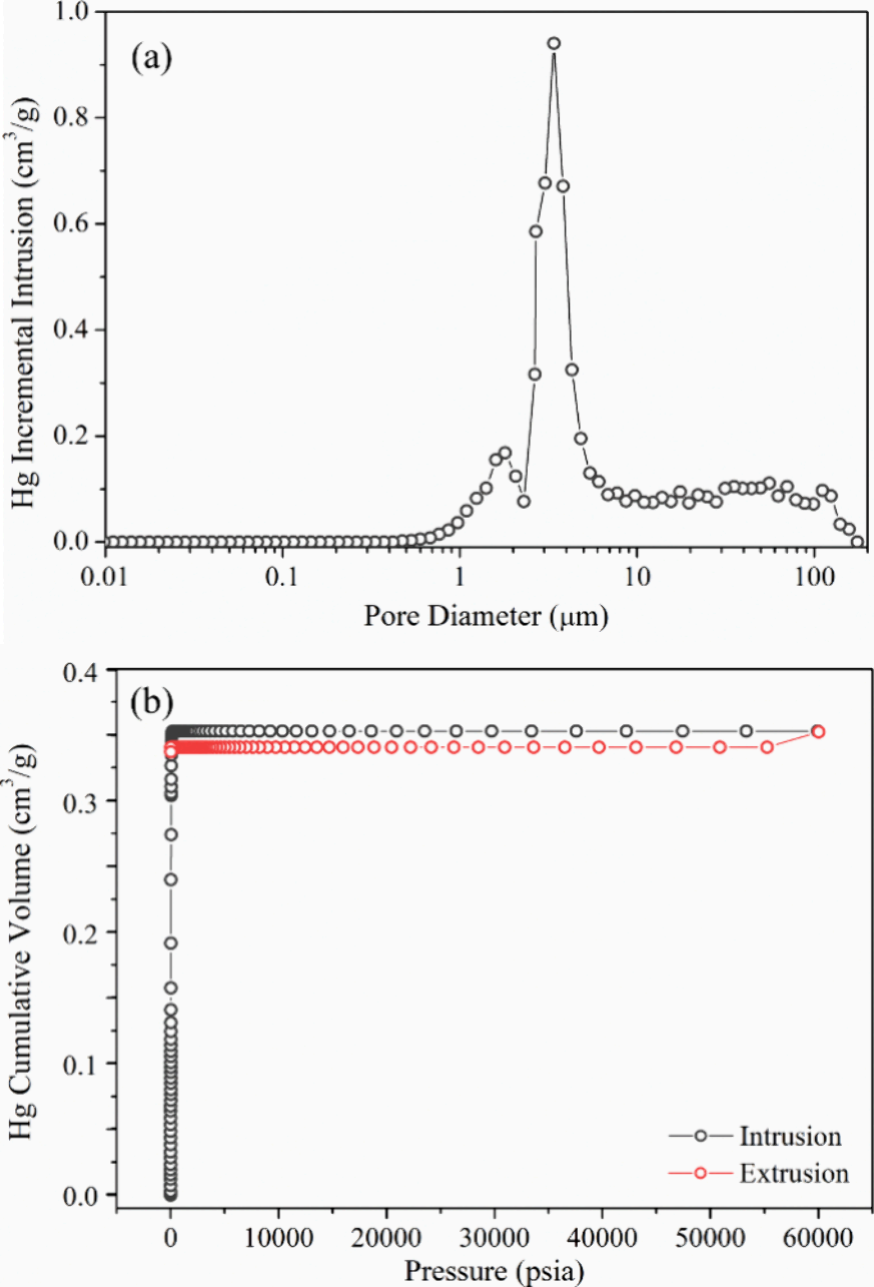
Mercury intrusion porosimetry findings for sintered SS 410S PMS samples. (a) Incremental pore size distribution curve, revealing a multimodal structure with dominant pore sizes of 1–6 μm and a secondary distribution from 10 to 100 μm interval. (b) Cumulative mercury intrusion and extrusion curves as a function of applied pressure, showing low hysteresis and good pore connectivity.

### 3.6 Degradation and Sintering Problems

As previously discussed, the metallic powders used in the PMS fabrication are composed of various alloying elements, with iron and chromium as the primary constituents. Chromium is added to enhance corrosion resistance by forming a stable, passive oxide film on the metal surface, especially in low-carbon steels exposed to oxidative environments such as atmospheric air.^59^ However, at elevated temperatures, chromium tends to diffuse from the surface into the bulk due to concentration gradients within the alloy. During this migration, Cr atoms can recombine with other elements and form precipitates, typically at grain boundaries.^60–62^ While these precipitates can contribute to improved mechanical strength under certain conditions, the formation of chromium carbides at high sintering temperatures can lead to embrittlement, reducing the alloys toughness and ductility.^63,64^

In the context of MS-SOFC applications, chromium diffusion and carbide precipitation are vital issues, as they can obstruct electronic pathways within the PMS structure, increasing ohmic resistance and compromising electrochemical performance.^6,65^ Therefore, controlling oxidation during sintering is critical to ensure optimal PMS functionality. A common strategy involves sintering in inert or reducing atmospheres using argon or nitrogen to prevent oxidation and hydrogen to reduce residual surface oxides formed during the burnout of organic additives at ambient conditions.

Figure 8a shows the surface microstructure of SS 410S PMS sintered at 1300 °C in an argon atmosphere. The grains exhibit a typical ferritic morphology characteristic of low-carbon stainless steel, with no visible signs of chemical or thermal etching. Notably, no feasible chromium carbide precipitates are observed under these conditions. In contrast, Figure 8b and c depicts the microstructure of samples sintered at the same temperature but in an ambient atmosphere, where numerous dark spots are evident along grain boundaries and triple junctions, suggesting the formation of precipitates. EDS analysis performed on one of these regions (Figure 8d) confirms the presence of chromium, carbon, and silicon, consistent with the feasible formation of chromium carbides. The spectrum reveals a higher atomic percentage of chromium (21.8%) compared to iron (5.0%), along with a significant oxygen and silicon content. The detection of silicon in these regions suggests that, at high temperatures, silicon from the SS 410S alloy may diffuse preferentially toward grain boundaries and triple junctions, where it tends to segregate due to the elevated interfacial energy. Such segregation and precipitation phenomena are known to contribute to embrittlement and can impair the electronic conductivity of the PMS structure.^66–69^

**Figure 8:**
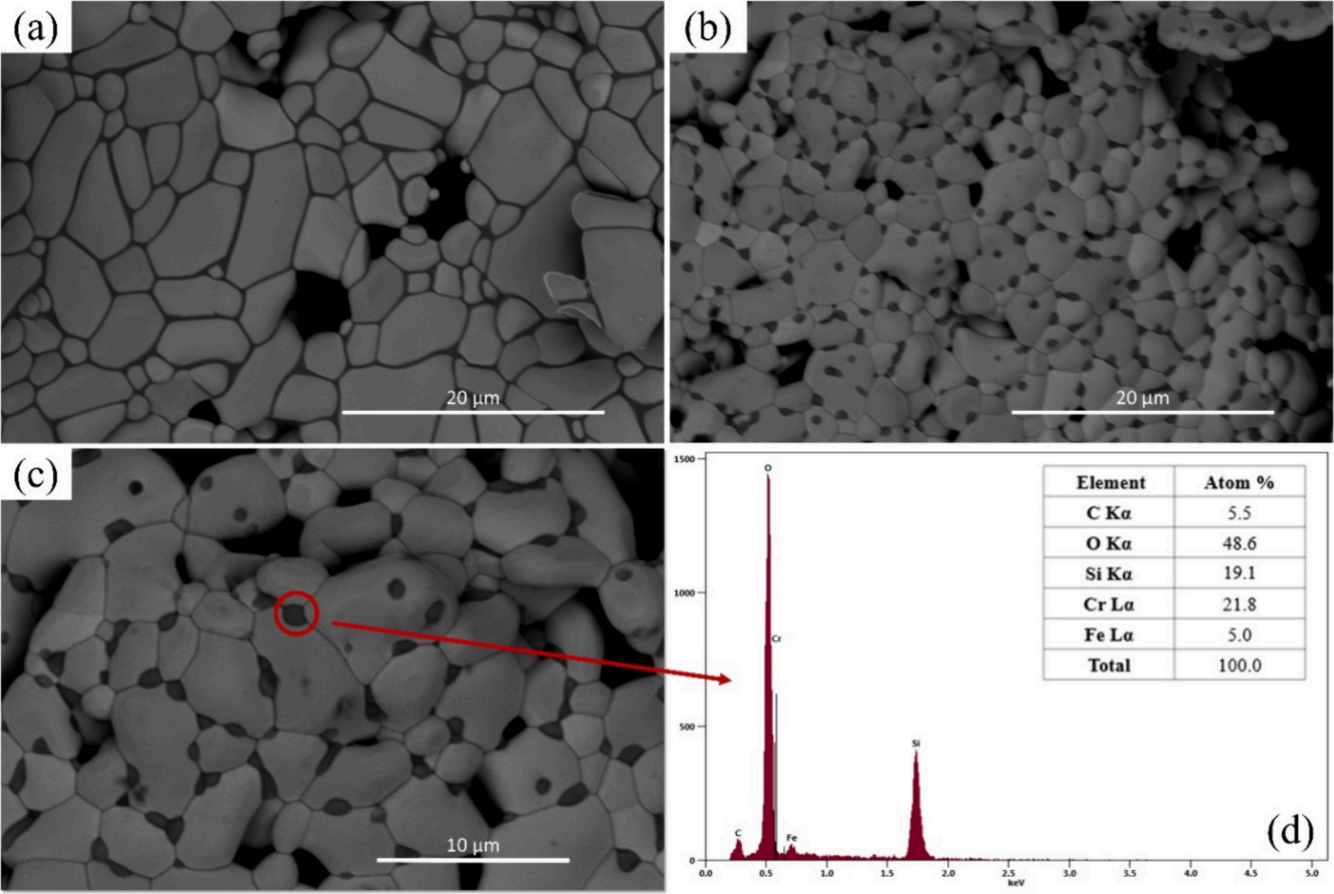
SEM and EDS analysis of SS 410S PMS sintered at 1300 °C. (a) Surface micrograph of the sample sintered in an argon atmosphere, showing well-defined grain boundaries and the absence of precipitates. (b, c) Surface micrographs of samples sintered in an ambient (oxidizing) atmosphere, revealing the presence of dark precipitates along grain boundaries and triple junctions. (d) EDS spectrum and corresponding atomic quantification of the region highlighted in (c), indicating the presence of Cr, Si, and C, which is consistent with feasible chromium carbide and silicate precipitates.

Figure 9 depicts the XRD patterns of the SS 410S PMS samples sintered at 1300 °C for 2 h under ambient air (pattern **a**) and argon atmosphere (pattern **b**). In both cases, partial oxidation of the PMS is observed, but the extent differs significantly. The sample sintered in air shows extensive oxidation, with diffraction peaks primarily corresponding to Fe_2_O_3_ (ICSD PDF #01–084–0308) and Cr_2_O_3_ (ICSD PDF #01–084–0314),^70^ indicating near-complete transformation of the metallic phase into oxides. According to Xu and Zhu,^71^ the presence of overlapping peaks in this region may also suggest the formation of a mixed oxide phase or solid solution composed of (Fe,Cr)_2_O_3_. The corresponding optical image reveals a characteristic rust-orange coloration, typical of oxidized ferritic steels, confirming the degradation of the Fe-based metallic matrix.^72,73^ In contrast, the XRD pattern of the sample sintered in argon exhibits α-Fe­(Cr) as the dominant phase (COD PDF #96–152–3983),^74^ with only minor signals from Fe_2_O_3_ and Cr_2_O_3_, indicating limited oxidation. EDS elemental mapping (Figure S4) confirmed the presence of iron, chromium, and oxygen on parts of the metallic support surface, even in an argon atmosphere. This preservation of the metallic phase is also reflected in the dark-gray appearance of the sample in the optical image. These findings align with previous reports in the literature, which highlight the effectiveness of inert atmospheres in minimizing oxidation during high-temperature sintering processes.^71,75–77^

**Figure 9:**
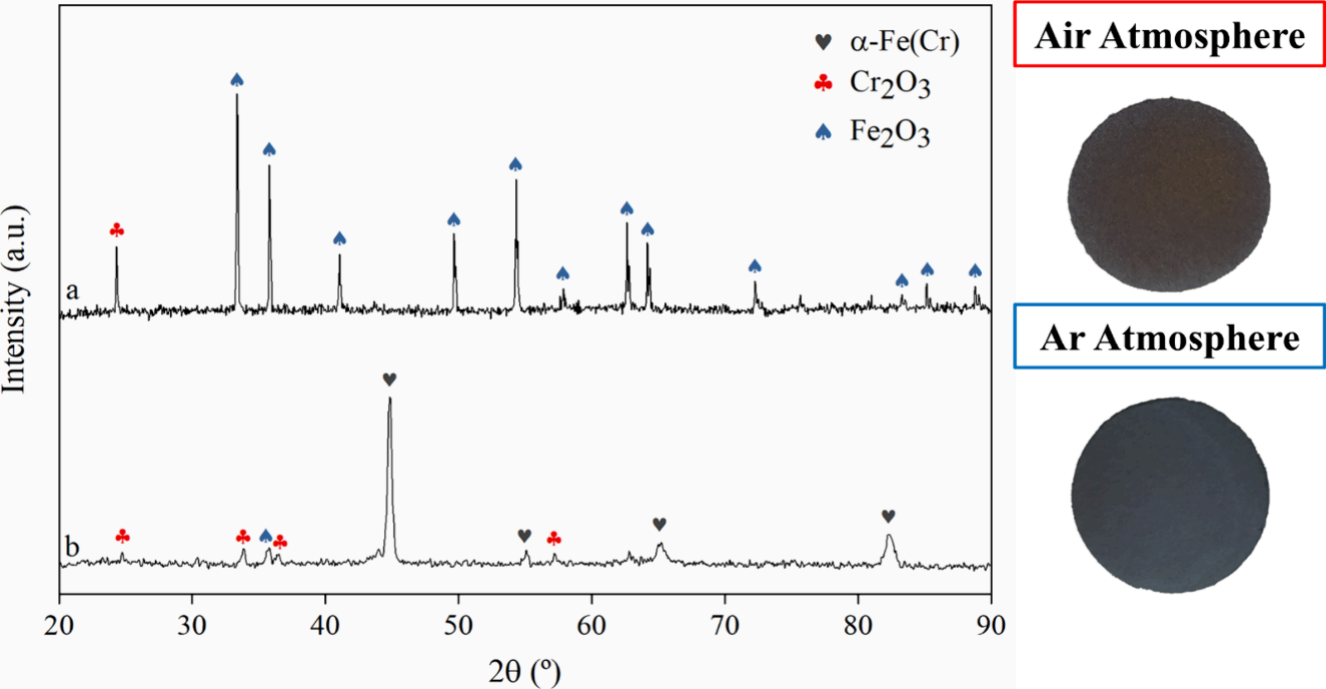
XRD patterns and corresponding optical images of SS 410S PMS samples sintered at 1300 °C for 2 h under different atmospheres: (a) air and (b) argon. The sample sintered in air shows predominant peaks of Fe_2_O_3_ and Cr_2_O_3_, indicating extensive oxidation, while the sample sintered in argon retains α-Fe­(Cr) as the main phase, with minimal oxide formation. The optical images corroborate the XRD results, with the air-sintered sample exhibiting a rust-colored surface and the argon-sintered sample maintaining a metallic gray appearance.

In summary, although the sintering process was conducted under an inert gas flow rate, slight oxidation of the PMS samples was still observed. This can be attributed to residual oxygen adsorbed on the surface of refractory materials, trapped within the chamber, or present as trace contaminants in the gas supply itself.^78^ This phenomenon is described in the literature by Reisert et al.^77^ as the formation of chromia scales. According to their findings, mild oxidation at elevated temperatures, whether during sintering or early stages of operation, can actually be beneficial for MS-SOFC applications. Such initial oxidation stabilizes the surface and suppresses further oxide layer growth at lower temperatures. In more critical scenarios, an effective strategy to further mitigate oxidation was reported by Fu et al.^78^ The authors demonstrated that combining an inert/reducing atmosphere with titanium pieces inside the furnace chamber can effectively scavenge residual oxygen. Due to titanium favorable oxidation thermodynamics, it reacts preferentially with oxygen to form TiO_2_, thereby serving as a sacrificial getter material. This reaction reduces the local oxygen partial pressure and prevents oxidation of the Fe–Cr alloy, offering enhanced protection of the PMS structure during thermal processing.^78^

### Electrical, Mechanical, and Permeability Characterization

Figure 10 shows the *I*–*V* characteristics of different SS 410S PMS measured using a parameter analyzer. All samples were measured with a single electrical contact placed at the central region (Figure S5). The characterization revealed a linear ohmic behavior in the voltage range between −0.4 V and +0.4 V for the PMSs. The conductance of each sample was obtained from the slope of the linear region of the corresponding curve, as shown in Figure 10. The resistance and resistivity of the PMSs were determined from the inverse of the conductance and the geometric area of the electrical contact. The intercepts did not affect the evaluation of the results, as they presented very small values, most likely corresponding to noise measurement.

**Figure 10:**
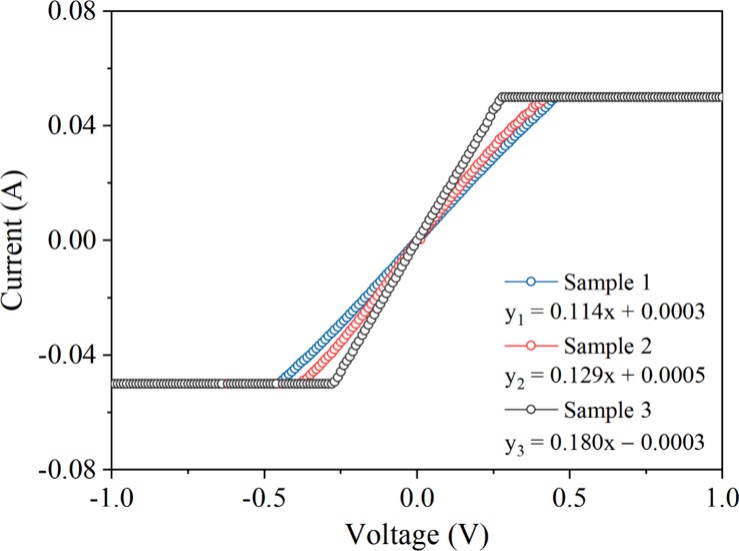
*I*–*V* characteristics of three different SS 410S PMS samples.

The data presented in Table 4 show that the conductance of the SS 410S PMS samples ranges from 0.114 to 0.180 S, indicating minor differences in the electrical current-carrying capacity among the samples. The resistance, inversely proportional to the conductance, varies between 5.54 and 8.75 Ω, reflecting the same trend observed in the resistivity measurements, which range from 2.19 to 2.75 Ω cm. The calculated electrical conductivity shows a gradual increase from samples 1 to 3, ranging from 0.364 to 0.457 S.cm^–1^, confirming the inverse relationship between resistance and conductivity. The mean values and standard deviations indicate good consistency among the samples, with relatively small variations. These results suggest that the PMS processing enables homogeneous electrical properties, suitable for applications requiring stable conductivity.

**Table 4:** Electrical Parameters Obtained from *I*–*V* Curves of Three Different SS 410S PMS Samples

**sample**	**conductance (S)**	**resistance (Ω)**	**resistivity**(Ω cm)	**conductivity** **(S cm** ^**–1**^ **)**
sample 1	0.114	8.75	2.75	0.364
sample 2	0.129	7.78	2.42	0.413
sample 3	0.180	5.54	2.19	0.457
average	0.141 ± 0.035	7.36 ± 1.64	2.45 ± 0.28	0.411 ± 0.047

Figure 11 compares the mechanical properties of PMS samples sintered in air and Ar atmospheres. The load–displacement tests demonstrated that sintering in air produced a brittle microstructure with low mechanical strength, reaching the elastic limit at 1.28 N (region A) and failing prematurely at 0.43 mm. In contrast, sintering under Ar resulted in significantly improved properties. The PMS sintered in an inert atmosphere exhibited an elastic limit of 3.95 N (region B), showing 3.1 times higher strength in the elastic regime compared to the PMS sintered in an oxidizing atmosphere, and showed greater ductility (3.6 times more ductile), with fracture occurring at a displacement of 1.55 mm. It can be concluded that the inert atmosphere is essential to preserve the 410S microstructure, providing the strength and ductility required for the PMS.

**Figure 11:**
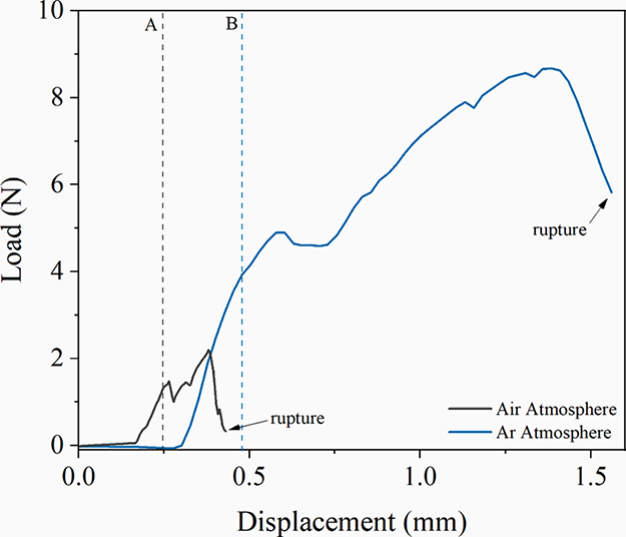
Load–displacement curves of PMSs sintered in an oxidizing atmosphere (black line) and an inert atmosphere (blue line), obtained via an adapted mechanical test until PMS rupture.

The gas permeability tests for the SS 410S PMS were performed using a H_2_ flow rate of 200 mL min^–1^. The permeability was calculated according to Darcy’s law, and values ranging from 5.85 to 8.36 × 10^–12^ m^2^ were obtained for three different SS 410S PMS samples. Additionally, the SS 410S PMS developed in this work exhibited superior permeability compared with other porous metallic substrates reported in the literature (Table 5). It is worth noting that the permeability to H_2_ was approximately 2.5 times higher than the permeability measured with liquid mercury by mercury intrusion porosimetry.

**Table 5:** Comparison of Gas Permeability with Metallic Substrates Reported in the Literature

**material**	**permeability** **(10** **^–12^** **m** ^**2**^ **)**	**porosity (%)**	**gas inlet**	**ref.**
SS Martensitic	4.05	19.9	nitrogen	^79^
AiSi10Mg	4.40	34.7	nitrogen	^80^
SS 316L	4.20	42.4	nitrogen	
Inconel 625	1.30	33.1	nitrogen	
Ti-6Al-4V	2.10	33.8	nitrogen	
Mold Steel 1.2709	0.35	30.0	air	^81^
Ti–48Al–6Nb	6.16	52.0	nitrogen	^82^
SS 410S	8.36	66.5	hydrogen	this work

### Overview and Comparison with Other Metal Supports Manufactured by Tape Casting

As previously discussed, the fabrication of PMS by aqueous tape casting remains a significant challenge, and consequently, only a few studies addressing this topic can be found in the literature.^21,22,30,31,83–87^ Furthermore, the proper physicochemical characterization of these components is often neglected. As shown in Table 6, only a limited number of processing and performance parameters have been reported. Therefore, this study aimed to fill some of the existing gaps in the literature. The PMS substrates developed for MS-SOFCs via aqueous tape casting in this work demonstrated performance comparable to, or even superior to, those previously reported.^30,31,86^ Specifically, a permeability 15 to 25 times higher than that reported in other studies was achieved,^21,86^ primarily due to the high porosity obtained. In addition, the mechanical strength of the developed PMS was found to be comparable to those of other PMSs reported in the literature.^83^ No isolated values of resistivity or conductivity for other substrates were found for direct comparison.

**Table 6:** Characteristics of Metallic Supports for MS-SOFC Reported in the Literature

**metal**	**solvent**	**porosity (%)**	**permeability** **(10** **^–12^** **m** ^**2**^ **)**	**maximum load (N)**	**conductivity** **(S cm** ^**–1**^ **)**	**ref.**
NiFe alloy	toluene/ethanol			1.9–3.9		^83^
Fe22Cr	methyl ethyl ketone/ethanol	40–50				^22^
NiFe alloy	xylene/ethanol	40–62				^84^
430L	*N*-methyl-2-pyrrolidone	44–51	0.37–0.58			^21^
430L	toluene/isopropanol	15–35				^85^
430L	water	10–45	0.31			^86^
410S	water	66.5	5.85–8.36	3.95	0.411	This Work

## 4 Conclusions

In this work, PMSs based on SS 410S were successfully fabricated via aqueous tape casting as a scalable and cost-effective approach for MS-SOFC applications. The alloy was selected due to its CTE compatibility with ceramic components and its inherent corrosion resistance, supported by a chromium content between 11.5% and 14.5%. The formulation of the slurry was optimized to yield flexible, defect-free green tapes. The final suspension, containing 34.9 vol % SS 410S, 18.3 vol % binder, 7.6 vol % cobinder, and 16.5 vol % plasticizer, exhibited pseudoplastic and thixotropic behavior with excellent stability, recovering 91.5% of its initial viscosity in the 3iTT test. TGA enabled the precise definition of the debinding profile, minimizing defect formation during burnout. Sintering trials conducted in air and argon atmospheres revealed a strong dependence on environmental conditions. Samples sintered in argon retained the metallic α-Fe­(Cr) phase and exhibited minimal oxidation, while those processed in air experienced extensive oxidation and chromium depletion, confirmed by XRD and EDS analyses. To prevent warping and chemical interactions with refractory supports, a precalcined ZrO_2_ powder layer was applied over porous Al_2_O_3_ sintering supports. This strategy proved effective in avoiding surface contamination and preserving tape geometry. PMSs fabricated with this approach demonstrated a lamellar microstructure and high interconnected porosity (66.5%), both of which are advantageous for gas diffusion in SOFC operation. Despite the high porosity, the samples maintained sufficient mechanical integrity for handling, as validated through qualitative drop tests, an essential requirement for further layer deposition in MS-SOFCs. Moreover, PMSs showed good gas permeability and mechanical and electrical resistivity. Altogether, this study validates a robust fabrication route for SS 410S-based PMSs via aqueous tape casting, integrating material selection, rheological control, and atmosphere engineering to overcome critical challenges in support preparation. The proposed methodology lays the foundation for future studies on cell integration, long-term stability, and electrochemical testing in full MS-SOFC assemblies.

## Supplementary Material



## Notes

Supporting figures and tables: Permeability measurement setup; Components of the suspensions of PMS; Rheological behavior of PMS suspension used for Tape D; Effect of sintering in different conditions of PMSs from Tape D; Linear shrinkage of PMSs from Tape D; SEM micrographs and elemental mapping of oxidated region of PMS sintered in argon atmosphere; Electrical measurement setup (PDF)
