# Supplementary material for: Engineering Lamellar Stainless Steel 410S Porous Supports via a Water-Based Tape Casting Method: A Scalable Path for MS-SOFCs
Source: ACS Omega. 2025 Oct 29;10(44):52547–61. doi: 10.1021/acsomega.5c05721 (PMC12612980; doi:10.1021/acsomega.5c05721)
Supplement: Supplementary file 1 [file ao5c05721_si_001.pdf]

Supplementary Information

Engineering Lamellar Stainless Steel 410S Porous Supports via Water-based Tape Casting method: A Scalable Path for MS-SOFCs

João P. J. de Oliveira[1,*], Fabio C. Antunes[1,2], Victor C. Normandia[3], Thiago Dias[1,3], Reinaldo Cesar[1,2], Débora Vilela Franco[4], Leonardo Morais Da Silva[1,4], Gustavo Doubek[3,*], and Hudson Zanin[1,*]

[1] Advanced Energy Storage Division, Center for Innovation on New Energies, School of Electrical and Computer Engineering, University of Campinas, Av. Albert Einstein 400, Campinas, SP 13083-852, Brazil.
[2] Centre for Energy and Oil Studies, University of Campinas, Av. Cora Coralina 350, Campinas, SP 13083-896, Brazil.
[3] Advanced Energy Storage Division, Laboratory of Advanced Batteries (LAB), Center for Innovation on New Energies, School of Chemical Engineering, University of Campinas, Av. Albert Einstein 400, Campinas, SP 13083-852, Brazil.
[4] Department of Chemistry, Laboratory of Fundamental and Applied Electrochemistry, Federal University of Jequitinhonha and Mucuri's Valley, Rodovia MGT 367, km 583, 5000, Alto da Jacuba, 39100-000, Diamantina, MG, Brazil.

*Corresponding authors: João P. J. de Oliveira, Gustavo Doubek, and Hudson Zanin
E-mail address: joaojenson1@gmail.com, doubek@unicamp.br, and hzanin@unicamp.br

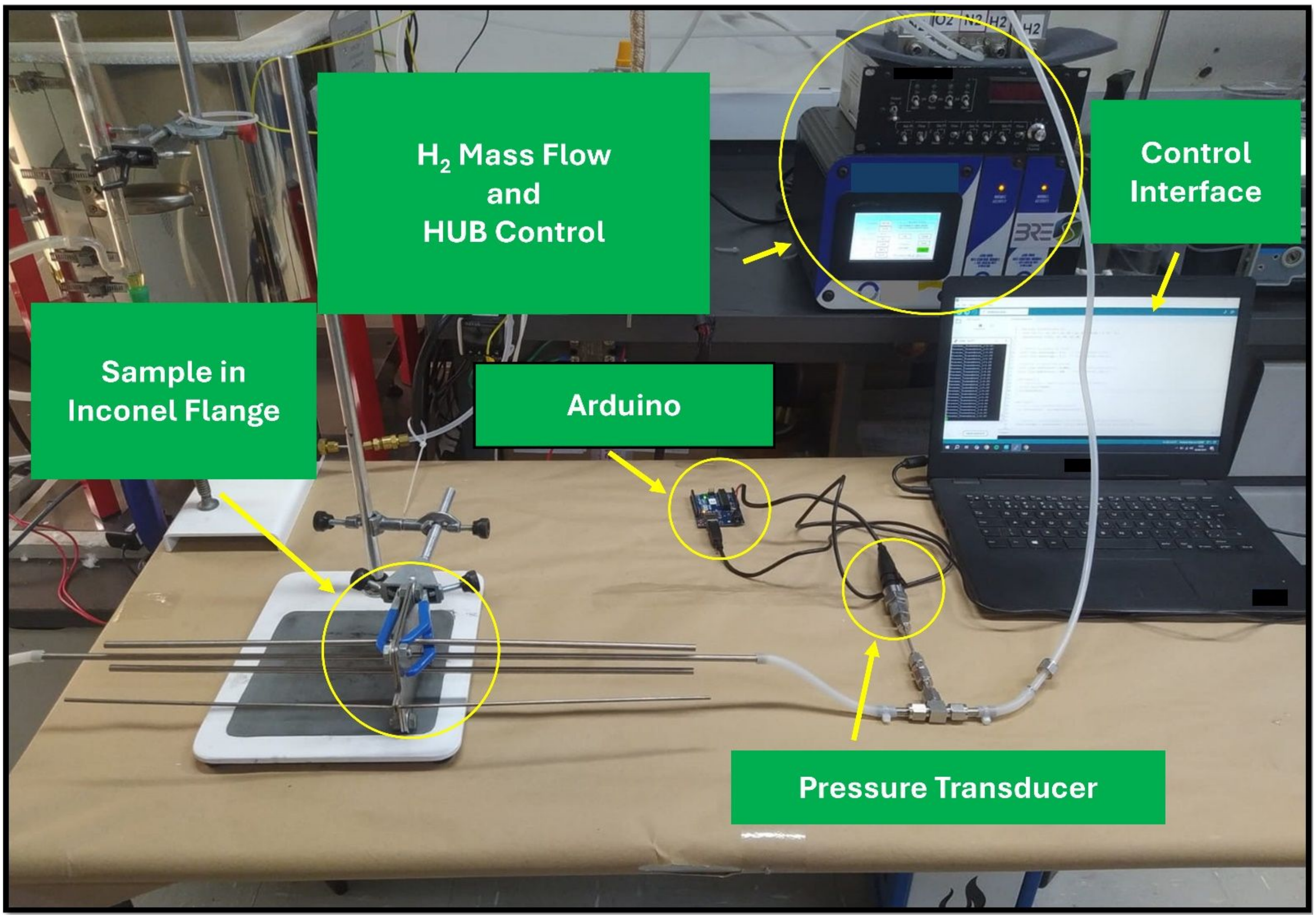

Figure S1. Permeability measurement setup.

Table S1. Values of the amount of each component of the suspensions referring to **Figure 2a–d**.

Component (%vol)	Tape A	Tape B	Tape C	Tape D
**Powder**	47.1	41.8	34.9	25.5
**Dispersant**	6.5	5.1	4.3	3.2
**Pore former**	12.2	12.5	11.7	10.2
**Binder**	11.9	15.6	18.3	22.5
**Co-binder**	4.9	5.2	7.6	11.2
**Plasticizer**	10.7	13.1	16.5	20.7
**Surfactant**	2.2	2.2	2.2	2.2
**Antifoam**	4.5	4.5	4.5	4.5

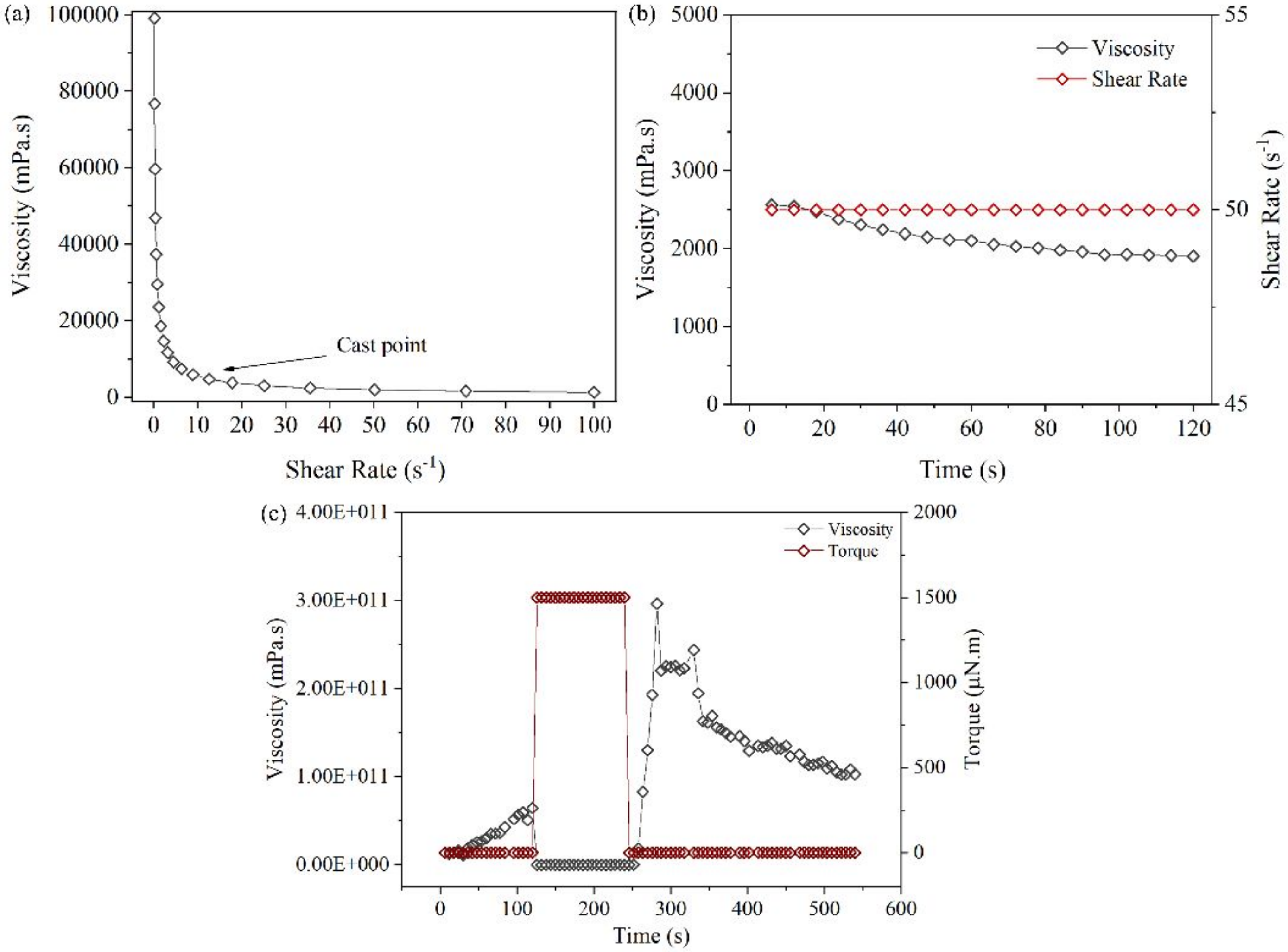

Figure S2. Rheological behavior of the optimized PMS suspension used for Tape D fabrication: (**a**) viscosity profile as a function of shear rate at 25ºC, showing pseudoplastic behavior and identifying the casting shear rate (~10 s^{-1}); (**b**) viscosity stability under constant shear rate of 50 s^{-1} for 120 s, simulating the tape-casting deposition time; (**c**) three-interval thixotropy test (3iTT), showing viscosity after structural breakdown caused by high shear torque (1500 µN m).

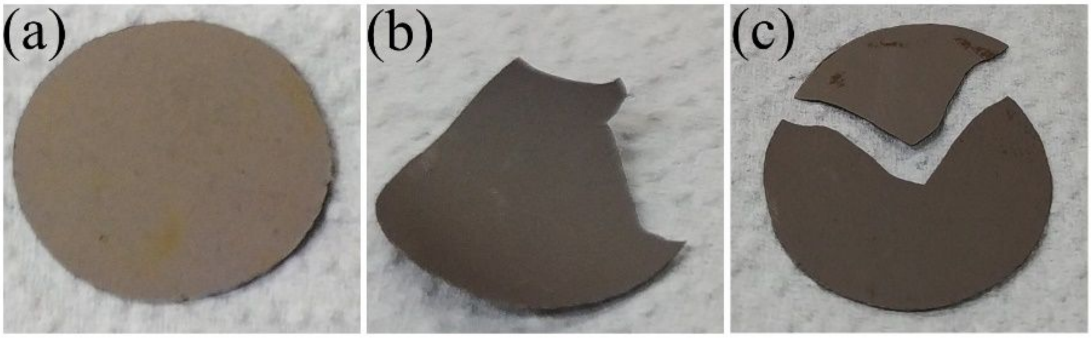

Figure S3. Photographs illustrating the effect of sintering conditions on PMS samples: (**a**) PMS sample sintered between refractories with a pre-calcined ZrO_2 powder, showing no visible warping or contamination, confirming the effectiveness of the protective layer. (**b**) PMS sample sintered without refractories, showing visible warping. (**c**) PMS sample sintered between refractories without a pre-calcined ZrO_2 powder, showing fractured caused by contact between sample and refractories.

Table S2. Linear shrinkage parameters of PMS samples of Tape D sintered at 1300ºC in argon atmosphere with sintering load plus pre-calcined ZrO_2 powder layer. Values represent average shrinkage percentage in diameter and thickness directions of 3 different PMS samples (*n* = 3 samples).

**Parameters (*n* = 3 samples)**	**Average Size with sintering load and pre-calcined ZrO_2 powder layer**	**Shrinkage with sintering load and pre-calcined ZrO_2 powder layer (%)**
**Diameter**	24.4 ± 0.19 mm	13.9 ± 0.31
**Thickness**	465 ± 12.5 µm	21.6 ± 2.0

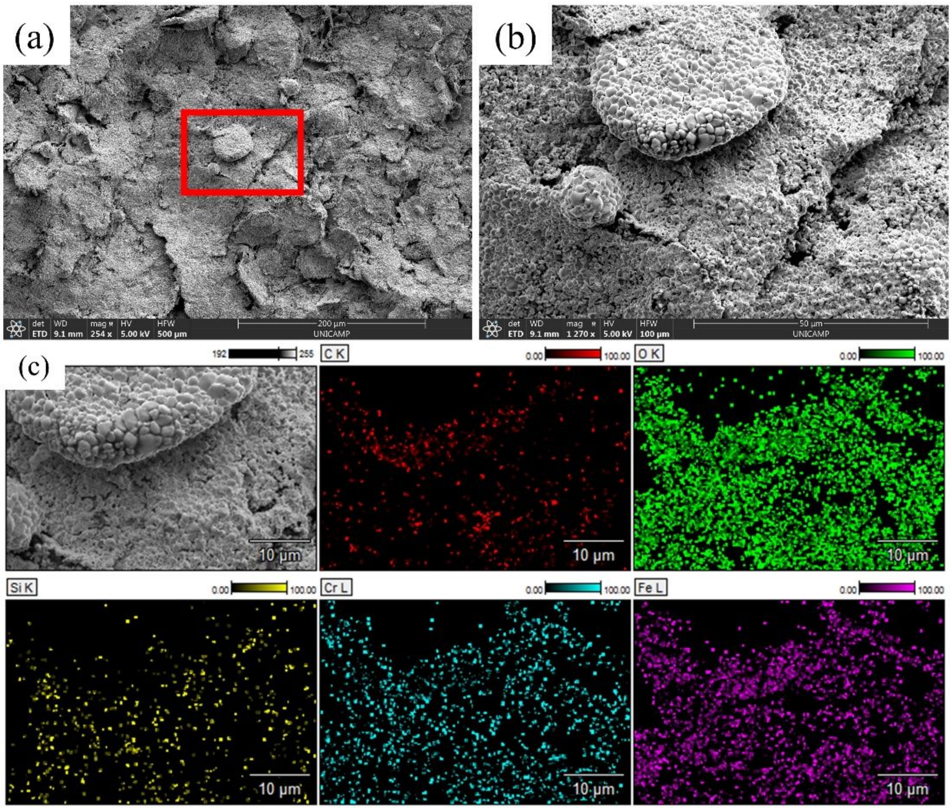

Figure S4. SEM micrographs of region of PMS SS 410S sintered at 1300ºC for 2 h under an argon atmosphere in different magnification of HFW: 500 µm (**a**) and 100 µm (**b**). (**c**) Elemental mapping analysis of zoom of region from **Figure S3a–b** showing Fe, Cr and O.

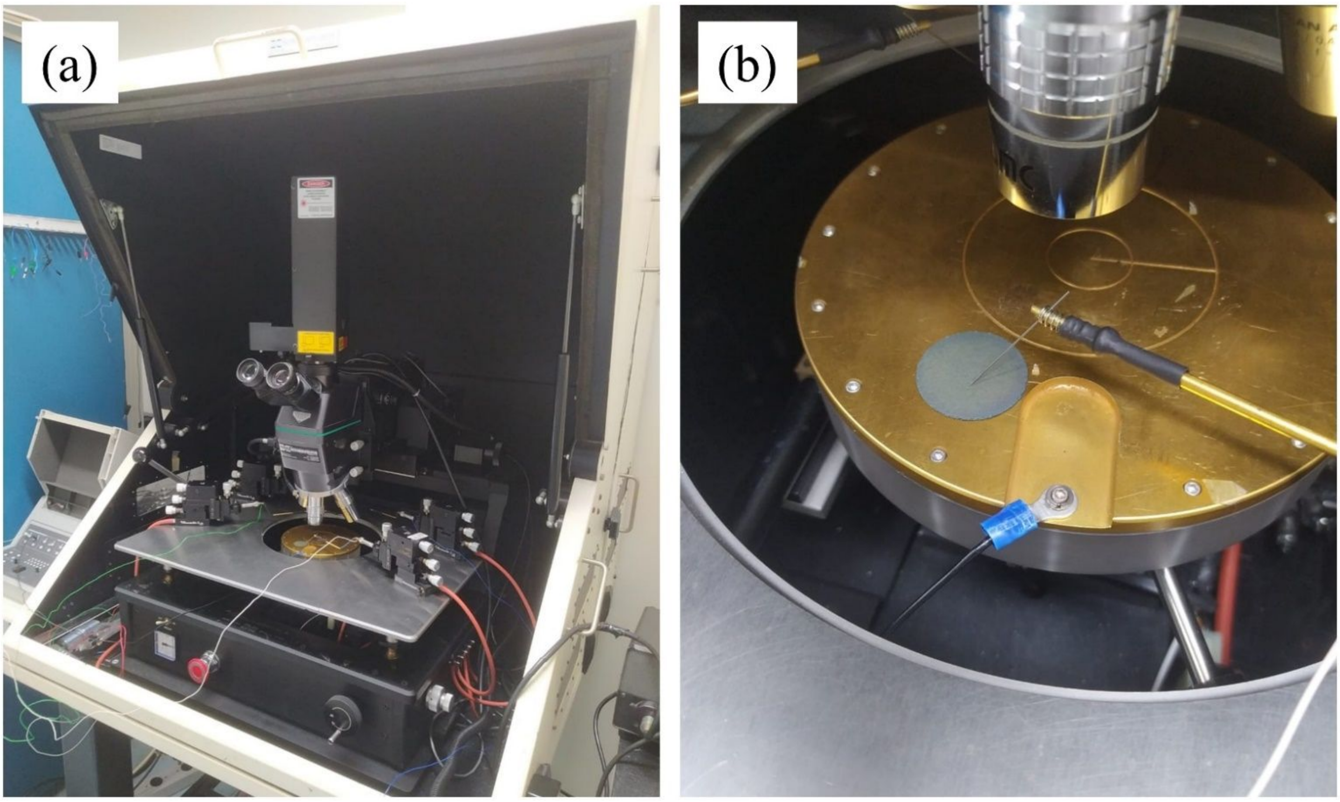

Figure S5. (**a**) Parameter analyzer Keithley 4200-SCS using to perform *I–V* measurements of PMS 410 S. (**b**) PMS 410S centralized in a parameter analyzer.
